# Climate finance and the legitimacy machine: Insights from the Green Climate Fund in South Africa

**DOI:** 10.1177/25148486261433439

**Published:** 2026-03-26

**Authors:** Jonathan Barnes, Richard Perkins

**Affiliations:** 1 Department of Risk and Disaster Reduction, 4919 University College London, London, UK; 2 Department of Geography and Environment, 4905 London School of Economics and Political Science, London, UK; 3 Centre for Economic Transition Expertise (CETEx), London School of Economics and Political Science, London, UK

**Keywords:** Assemblage, climate finance, Green Climate Fund, legitimacy, South Africa, transformation

## Abstract

The Green Climate Fund (GCF) is the principal multilateral mechanism for channelling climate finance to developing countries. This paper examines the processes through which GCF projects are developed, opening the ‘black box’ of project development to better understand the uneven production of different kinds of climate finance. Empirically, the analysis addresses a puzzle in South Africa: while the Development Bank of Southern Africa (DBSA) had secured approval for three projects by 2019, it was not until 2025 that the South African National Biodiversity Institute (SANBI) secured approval for its first project. To account for this divergence, the paper brings assembling thinking into dialogue with the concept of legitimacy. A key contribution is to introduce and operationalise the ‘legitimacy machine’ to theorise how assemblages become productive. This abstract machine transforms disparate components into sufficiently legitimate entities to advance policy objectives. Legitimacy thus acts as a mechanism for establishing and strengthening connections, unlocking relational power, and enabling the flow of authority, resources, and material effects. The paper demonstrates how the production of climate finance in South Africa reflects contrasting processes of legitimacy-making in the pursuit of heterogeneous desires. DBSA rapidly mobilised private finance by legitimating the South African economy as an investible opportunity, while shielding projects from critique through decentralised delivery. SANBI, by contrast, deliberately pursued a slower, more inclusive approach – shaped by acute scarcity of adaptation finance and the political challenge of narrowing multiple project ideas without alienating ‘deserving’ publics. Its commitment to safeguarding its own institutional legitimacy outweighed the imperative of rapid approval. These findings nuance debates on the transformational potential of the GCF and its role in the top-down financialization of recipient countries. They also underscore the need for pragmatic and differentiated approaches to supporting projects, recognising that distinct forms of multilateral finance require both rapid and patient pathways.

## Introduction

A defining feature of the international climate regime in recent decades has been calls to accelerate climate finance to the Global South. The commitment by developed countries to mobilise US$100 billion per year for adaptation and mitigation projects in developing countries by 2020 was a significant – albeit insufficient – milestone in the response to these demands (Browne, 2022; Kalinowski, 2024). Operational since 2015, the Green Climate Fund (GCF) serves as the primary global mechanism for channelling this climate finance.

Given the GCF's pivotal role in distributing public climate finance, it is crucial to understand how and why certain kinds of climate-related projects are proposed and ultimately approved. Responding to calls for deeper insight into the factors shaping GCF project proposals (Kuhl and Shinn, 2022: 1301), this paper examines project development within recipient countries. Specifically, we ask: beyond capacity constraints, what shapes (1) why some project proposal processes progress while others stall, and (2) why certain types of projects are more readily initiated than others?

To explore these questions, we focus on an empirical puzzle from South Africa. The country has two contrasting direct access entities (DAEs) – national organisations accredited to develop GCF funding proposals and receive finance directly. Direct access is of particular significance because, in theory at least, it is more likely to reflect national and local visions of transformation and self-determination (Barnes, 2022b; Johnson et al., 2022). Several projects developed by the Development Bank of Southern Africa (DBSA) were approved relatively quickly, while a single adaptation project developed by the South African National Biodiversity Institute (SANBI) took many years to secure approval.

To better understand these divergent experiences, we integrate insights from two strands of literature: assemblage and legitimacy. Assemblage thinking emphasises contingency, process, and becoming, rather than merely outcomes (Buchanan, 2017; Savage, 2020). It is therefore well suited to analysing the complexity, competing agendas, and ‘messiness’ of project development, including the often-overlooked work that precedes formal project documents. We complement this with the concept of legitimacy, which offers valuable insight into the relational dynamics within assemblages. By foregrounding questions of appropriateness and authorisation, legitimacy helps explain why certain relationships enable flows of authority, resources, and influence, while others do not. In this way, legitimacy sheds light on the uneven productivity of project development processes, and what ‘kinds’ of climate finance are enacted in practice (Bryant and Webber, 2024).

This paper makes two contributions. First, we advance the literature on international climate finance by providing fresh insights into the challenges of disbursing public funds and initiating projects (Bracking and Leffel, 2021; Christophers, 2022). Our work complements – and moves beyond – existing studies of the GCF, which have largely focused on project documents (Kuhl and Shinn, 2022), reported results (Puri et al., 2022), and policies (Omukuti et al., 2022). While these studies highlight the GCF's procedural biases and the limitations of its transformational potential in terms of resilience, self-determination and depth of change, they often overlook the strategies, practices, and experiences of project developers. By analysing the development process itself, we nuance prevailing critiques of the GCF's transformational character. Our findings show that actors may be pursuing deep transformations (Kasdan et al., 2021; Kuhl et al., 2024) that are invisible if analysis is confined to publicly available data. Examining project development also reveals the domestic politics shaping GCF engagement – specifically, whose values, interests, and consent influence the programming of climate finance (Bigger and Millington, 2020; Thomas, 2024).

Second, we make a theoretical contribution by bringing together assemblage and legitimacy – two concepts that have largely remained separate in the literature. This allows us to make visible the dynamics of relational power within policy assemblages. We introduce and operationalise the *legitimacy machine* to theorise how desires transform components, and their configurations, into sufficiently legitimate forms that enable progress toward an assemblage's goals. We argue that legitimacy unlocks relational power, “creating the conditions of possibility for certain policies to emerge” (Savage, 2020: 331). Simultaneously, our analysis unsettles binary or deterministic understandings of legitimacy by showing how legitimacy considerations may impede assemblages, creating ‘unruly’ entanglements. In doing so, we offer a political and strategic reading of legitimacy attentive to the contingent nature of its effects *in situ*.

## Background

### The Green Climate Fund

Established under the United Nations Framework Convention on Climate Change, the GCF is the world's largest multilateral climate fund. Its mandate is to support developing countries in achieving “low-emissions, climate-resilient pathways” in fulfilment of their Paris commitments (GCF, 2025b). Using a range of instruments – grants, loans, guarantees and equity – the GCF channels funds for mitigation and adaptation projects. Although capitalised from public sources, the Fund seeks to crowd in private financing by de-risking investments.

The GCF purports to take a country-driven approach that aligns financing with domestic priorities. It does not develop and implement projects itself but relies instead on a distributed network of accredited entities to do so. Since 2015, the Fund has committed US$19.3 billion to 336 projects. By the end of 2024, 54 per cent of its funding portfolio in nominal terms was directed to mitigation and 46 per cent adaptation (GCF, 2025a).

At the outset, the GCF was invested with hope that it would usher in a more progressive approach to international climate finance emphasising country ownership, structural change and climate justice (Bryant and Webber, 2024). It is against this backdrop that subsequent critique has emerged. Some of the most vulnerable countries have not received GCF funding (Garschagen and Doshi, 2022). Despite the emphasis on local ownership, direct access to the Fund has proved particularly challenging. Only a comparatively small number of eligible countries – notably those with higher institutional capacity – have had domestic entities accredited (Garschagen and Doshi, 2022). Moreover, DAEs have not always had the capacity to engage with local stakeholders to understand their needs, or develop high-quality proposals (Fonta et al., 2018; Omukuti et al., 2022). As such, a significant amount of GCF financing has been programmed by international entities removed from domestic ownership (Zamarioli et al., 2020).

Scholarly critique has focused on the Fund's ability to bring about ‘transformative change’ – a political concept whose meaning remains subject to ongoing disagreement (Kalinowski, 2024; Kuhl et al., 2024). Within the GCF's own investment criteria, this is operationalised as ‘paradigm shift potential’, defined as the “[d]egree to which GCF can achieve sustainable development impact beyond a one-off project or programme investment through replicability and scalability” (GCF, 2025c). Analysing the creation of the Fund, Bracking (2015) argues that the concepts of transformative change and paradigm shift were co-opted by powerful interests as empty signifiers to further entrench neoliberal environmental governance. Likewise, focusing largely on board meeting reports, Bertilsson and Thörn (2021: 423) show how these concepts have been “used as discursive resources…to legitimize a top-down financialization of recipient countries, while describing it as country ownership and responsiveness to their needs.” Kuhl and Shinn (2022) study direct access proposals for approved GCF adaptation projects. They find that transformation potential in project documents is predominantly framed in terms of scalability and replicability, with the ‘breadth’ and ‘speed’ of transformation prioritised over ‘depth’ in terms ‘local engagement and capacity building’ (p. 1300).

Together, these studies offer a critique which suggests that, rather than deeply embedding transformative climate justice, the GCF risks reproducing dominant modalities of climate finance. These modalities privilege actors with existing access to finance, market-oriented logics, the interests of private capital and large-scale projects (Bracking and Leffel, 2021; Bryant and Webber, 2024; Treichel et al., 2024). Our point of departure is to move away from an analysis of descriptive statistics and documents submitted to the GCF by focusing on the contingent and messy work of project development. In doing so, we offer a nuanced interpretation of how two very different DAEs sought to use GCF resources, and what this says about the transformative potential of international climate finance.

### South Africa

South Africa makes a fitting context to explore GCF project development. It has an acute need for international climate finance for mitigation and adaptation – reflecting a heavy reliance on fossil fuels, significant growth in energy demand, and vulnerability to the impacts of climate change (Neumann, 2023). Poverty, inequality and the legacy of apartheid also mean that social justice issues loom large, with a vibrant civil society frequently (re-) politicising issues of energy, development and climate change (Barnes, 2025; Büscher et al., 2024; Phillips and Petrova, 2021). Unlike many sub-Saharan African countries (Fonta et al., 2018), South Africa has multiple DAEs accredited to the GCF, making comparative analysis possible.

The two contrasting DAEs examined in this paper were accredited to the Fund in 2016 (Table 1). DBSA is a state-owned enterprise with a broad mandate across Africa in multiple sectors. SANBI is a quasi-governmental organisation focused on local and ecosystem-based adaptation. The divergent experiences of these entities represent a rich opportunity to learn about the influences shaping direct access.

**Table 1.** Accredited entity comparison.

	Development Bank of Southern Africa (DBSA)	South African National Biodiversity Institute (SANBI)
Entity type	Direct (regional)	Direct (national)
Description	Government-owned development finance institution with particular focus on infrastructural planning and development	National public body with a mandate for biodiversity research and information dissemination. It also develops and implements climate resilience projects
Size	Large (total projected costs exceed US$250 million)	Small (total projected costs between US$10–50 million)
Types of financial instrument	Basic, project management, grant award, on-lending/blending, loans, equity, and guarantees	Basic, project management, and grant award
Thematic focus	Adaptation and mitigation	Adaptation
Sector	Public and private	Public

Source: Authors, drawing on information sourced from GCF (n.d.-b).

An important distinction between the entities relates to the end use of finance. The DBSA sought to promote green private sector activities by blending GCF money with other credit lines to improve the terms of loans. Against a backdrop of considerable need and scarcity of adaptation finance, SANBI sought grants to assist public sector institutions to manage environmental issues and benefit local communities. These different end uses are expressive of different normative futures being imagined for climate finance – one rooted in the green economy and another in climate justice (Bryant and Webber, 2024). They also reveal different legitimacy-granting audiences for the DAEs.

We explore how each DAE went about project development. The DBSA worked expediently throughout the process and, at the time of the research, had three projects approved.^1^ SANBI took a slower, more inclusive approach, developing ideas into concept notes^2^ and had a project approved in July 2025, when this paper was under review. Both DAEs have been criticised: the DBSA for a lack of consultation and its financing of coal, for example, and SANBI for the pace of its project development.

## Theoretical framework

In this section, we elaborate how assemblage thinking provides the conceptual tools to unpack the experiences of each DAE and explain their varied productivity.

### Assemblage

Assemblages can be interpreted as working arrangements of human and non-human components. They are productive, operating as ‘machines’ (Deleuze and Guattari, 1988), with relations between the components generating ‘new territorial organisations, new behaviours, new expressions, new actors and new realities’ (Müller, 2015: 29). Assemblages never fully fix, remaining immanent and in a constant state of becoming. There is always the potential for different outcomes, and this productive tension is maintained and explained by power relations between components of an assemblage. Assemblages may dissemble and unravel (‘de-territorialisation’) and disappear altogether but can also reassemble and stabilise (‘re-territorialisation’) (Briassoulis, 2019).

A wide range of socio-spatial formations – ranging from security regimes to hydropower projects (Ramos, 2022; Richmond, 2019; Vaishnava and Baka, 2022) – have been analysed using assemblage thinking. Within the present paper, assemblage is used to explore efforts to access the GCF, though it could equally be used to consider other sources of climate finance. We position the assemblage of interest as a policy assemblage (Savage, 2020). Policy assemblages unsettle notions of coherence, viewing policy as messy, unpredictable and provisional (Buchanan, 2017; Lea, 2020). Policy assemblages typically seek to control unruly and disruptive elements and bring these to productive order. This helps explain the momentum and throughput of an assemblage as it progresses towards its objective(s). GCF project development is usefully conceived this way as a series of flows of ideas and resources that seek coherence, order, and ultimately bankable projects.

Assemblages are strategic endeavours, deliberately transforming things into partially stabilised (‘territorialised’) formations (Savage, 2020; Ureta, 2015). Driving their productivity is desire, conceptualised ‘as an active, positive force’ (Haggerty and Ericson, 2000: 609). Central to the South African climate finance assemblage is the desire to channel resources from the GCF to South Africa and to channel results (i.e., emissions reductions, resilience, etc.) from South Africa to the GCF. An assemblage's purpose exists with reference to the strata, which are the multi-layered, historical formations ‘that give shape to daily life’ (Thompson et al., 2021: 9). The policy assemblage analysed in this paper draws components from the strata – comprising people, ideas and material things from both South Africa and the GCF – and orders desire to connect these via flows of finance and climate impact. This includes the desires of DAE staff, national policymakers, the private sector, staff at the GCF, as well as interested parties outside South Africa.

### Relational power and the legitimacy machine

An assemblage approach lends itself to analyses of political economy concerned with how particular arrangements come into being in certain places and times. It directs attention to configurations of power and who gets to shape outcomes through the ongoing work of assembling, coordinating and stabilising different elements (Li, 2007). Assemblage thinking theorises power as relational, contending that it is not held by actors or wielded over others. Rather, power is an immanent and emergent force enacted through relations between people and things (Lawhon, 2012). Powerful connections trigger flows in the assemblage, bringing it closer to its productive potential.

While offering insights into how power operates, this relational view fails to account for how connections are forged, stabilised and maintained. It also fails to account for why some connections become productive, while others remain unproductive. Indeed, by assuming ontological equivalence of everything, relational accounts risk depriving themselves of explanatory power (Tonkiss, 2011).

Against this backdrop it becomes useful to enlist other conceptual tools. Within policy assemblages, where consent, approval and authorisation may be necessary for ideas and resources to flow, we turn to the concept of legitimacy. Legitimacy – when ‘the actions of an entity are desirable, proper, or appropriate’ (Suchman, 1995: 574) – is fundamentally concerned with accepting authority and the rationale for doing so (Bernstein, 2011; Zelditch, 2018). It can be conceived as an inherently political condition predicated on the ability to justify action or acceptability (Clark, 2007; Stanley and Cusworth, 2025). This reading of legitimacy moves us away from binary conceptions where something is inherently legitimate or illegitimate based on the application of norms like legality or morality. It foregrounds the socially constructed nature of legitimacy, wherein legitimation (i.e., the process of acquiring or maintaining legitimacy) is a communicative process (Suddaby et al., 2017: 458).

Past assemblage work has not afforded legitimacy analytical primacy. Studies have focused on discursive and material techniques that are used to legitimate assemblages or their effects (i.e., outputs). For example, Nel (2017) explores how carbon forestry assemblages are made possible, highlighting ‘how appeals to scientific and market authority’ are enrolled to legitimate carbon forestry and offsets (p. 146). Adopting an assemblage approach, Li (2007), Kirkegaard et al. (2023) and Wissman et al. (2024) point to how various practices, procedures and devices are used to gain social acceptance, including by circumventing or silencing critical voices. These and cognate studies (Bracking, 2015; Fransen and Bulkeley, 2025; Kumar, 2021; Perkins, 2021) emphasise the construction of legitimacy and the ‘work’ involved in rendering certain arrangements legitimate. Assemblages involve a diversity of actors, desires, and approaches (Aijaz, 2023; Li, 2007). Which, and whose, legitimising practices and logics hold sway can have political (and anti-political) effects with material consequences for what is assembled.

Our novel contribution is to theorise legitimacy as connecting and sustaining component relations which can animate relational power and trigger effects. To do so, we draw on Deleuze and Guattari's (1988) diagrammatic mechanism of the ‘abstract machine’, comprising the network of conditioning relations which hold an assemblage together and give it purpose (Nail, 2017). Abstract machines provide the ordering logic for assemblages and can illuminate power relationships which lead to certain outcomes or frustrate others. In what follows, we invoke a particular type of abstract machine, the legitimacy machine. This conceptualises how certain policy assemblages legitimate things to effect change in the world. Specifically, we suggest that the legitimacy machine incorporeally transforms^3^ components, and configurations of components, to *become* recognised as legitimate. Incorporeal transformation refers to how, through the act of language, it is possible to alter a body's^4^ status or its relations to other bodies. An oft-cited example from Deleuze and Guattari's *Capitalism and Schizophrenia* is a judge passing sentence: the judge's words transform the body of the accused into that of a prisoner (1988: 96).

We develop the idea that GCF project development can be understood as a legitimacy machine. The policy assemblage is an ordering of desire which enrols and connects component parts and incorporeally transforms component relations into legitimate ones. This actualises relational power so that resources and ‘results’ can flow. The uneven production of legitimacy helps explain why, in some instances, the assemblage animates potentially powerful connections to become productive, while at other times it does not.

## Methods

The paper draws on fieldwork in South Africa between November 2018 and March 2020. A case-study research design (Thomas, 2016) enrolled multiple, interrelated communities with a vested interest in GCF programming. Initial field construction was built-out from the organisations formally involved in GCF project development, especially DBSA and SANBI. Memorandums of understanding were signed with each, facilitating insider access to staff, events, and written materials. Participation in forums, either arranged or attended by staff, furnished further insights. Multiple, often repeat, interviews were carried out with various representatives. In total, 80 semi-structured interviews were undertaken with relevant actors from the GCF, civil society (including communities and NGOs), labour unions, and the private sector. Documents formed part of the data for the analysis. Material not in the public domain was sought from the DAEs (e.g., internal minutes, process documents, etc.). This was supplemented by publicly available information (e.g., project proposals and meeting documents on the GCF website).

Data were analysed thematically (Braun and Clarke, 2021). Preliminary empirical inquiry focused on project development and the challenges faced by the DAEs in navigating contestation. Initial codes reflected constructs and ideas from the literature, such as strata, incorporeal transformation and flow. These were combined with empirical ideas like legitimacy to form initial ideas. This furnished higher-order codes and organisational themes before a second, more inductive and abductive round of coding.

## Assembling climate finance in South Africa

Using the analytic of assemblage, this section examines the DAE's experiences programming GCF resources with a view to explaining the puzzle of their contrasting productivity. Although each organisation also seeks access to other climate finance sources, our focus is on the desire to access GCF money. We take a chronological, synoptic perspective which covers three, overlapping sequential phases, as well as an additional phase when the desires ‘overflowed’ the boundaries of the assemblage (Figure 1). The periodisation facilitates a processual perspective on how legitimacy is produced and for whom, what flows are triggered, and instances when the productivity of the assemblage stalls. At each stage, we demonstrate how the legitimacy machine transforms components and relations between components and what effects this leads to.

**Figure 1.**
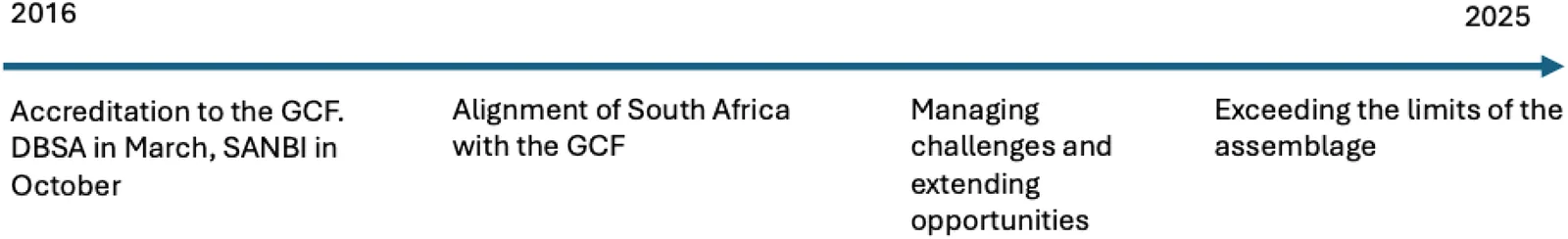
Phases of GCF project development within the assemblage. Source: Authors.

### Accreditation

The DAEs’ suitability as legitimate partners for the GCF were demonstrated by their existing characteristics and commitments to specific changes. Both were good candidates for direct access, with track records in programming climate finance. The GCF requires accredited entities to complete Accreditation Master Agreements (AMA) which codify responsibility and accountability. This process incorporeally transformed each DAE. In particular, it formalised the relationship between the DAEs and GCF into legitimate channels for the throughput flows of resources and trusted results. AMAs are generic documents, but their contents would preclude most organisations from participating. They detail the covenant of being an accredited entity. SANBI's AMA states that they must:

*…submit at least one periodic monitoring report for each of the projects… approved by the Adaptation Fund in order to provide evidence of the track record of SANBI in monitoring projects under its grant award mechanism* (GCF, 2017: 41)

The clause justified accreditation, based on its similar function with the Adaptation Fund. This legitimation is based on performance and the ability to report impacts of adaptation projects. It is almost circular: legitimacy accrued from one multilateral fund serves as an input to another. The international climate regime, of which the GCF is part, determines the type of organisations that can participate in climate finance. This reflects a “need for control and value for money” (GCF Board Member). The validation of another fund helped transform SANBI for the GCF. This is detached from SANBI's characteristics, staff, or civic legitimacy. It relied instead on track record, reproducing existing configurations of privileged access to climate finance. Since legitimation was based on SANBI's experience in local adaptation projects, it led to the expectation that GCF proposals should also be adaptation-focused and benefit the community, locking in specific pathways and audiences for legitimacy.

The DBSA went through the same legitimacy-granting accreditation process – again, demonstrating its existing credentials. For example, the proposal for a Climate Finance Facility (CFF) provided justification for DBSA's suitability as a DAE by strategically referencing experiences with other funds:

*The GEF*^5^ *DBSA portfolio is further proof that the Bank has a good track record for implementing projects such as the CFF. In terms the DBSA's recent track record with regards to sustainability measures, the DBSA continues to work towards embedding sustainability in its core business*. (GCF, 2018: 39)

In short, the DBSA sought to legitimate itself as a partner to the GCF through self-portrayal as a ‘safe pair of hands’, with ready-made partnerships and institutional capacity. There was also a commitment to improving, namely, by the greening of its ‘core business’. The rendering of the DBSA into a legitimate entity therefore was based on its existing attributes and commitment to change in the future. This partly reflects widespread concerns that the DBSA's management of the South Africa Green Fund was not “really looking for sustainable business models as much as they were interested in getting money out the door” (Climate and energy lawyer). The DBSA staff leading work on the CFF were aware of such criticism and that “the ability to measure impact is crucial to attract concessional funding”, so it is central to the DBSA's ambitions. The CFF materials commit to benchmark progress towards better environmental impact reporting via a Development Results Reporting Framework, which provided:

*…an accurate portfolio breakdown of the DBSA commitment to climate change thereby enabling credible and accurate reporting to stakeholders including the GEF, GCF and the International Development Finance Club.* (CFF project proposal: 41)

This asserts a ‘promissory legitimacy’ (Beckert, 2020) – in the sense of legitimacy gained from the credibility of future promises (p. 318) – that the DBSA sought to build via its CFF proposal. The DBSA's aim was partly to satisfy GCF's demands but also other audiences such as the International Development Finance Club.^6^ The CFF proposal had a performative effect, disseminating future achievements derived from GCF financing to justify the DBSA's credentials to wider legitimacy-granting audiences. SANBI meanwhile used accreditation to make concrete commitments to access socially just finance for South African beneficiaries.

The legitimacy machine conceptualises how the assemblage enlisted and legitimated the DAEs through accreditation. Accreditation brought the Fund and South Africa into a productive relationship whilst setting in motion specific potentially productive pathways. Accreditation enrolled the respective DAE with different components from the South African strata – such as the legacies of colonialism and apartheid, the national energy context and the financial constraints in the public and private sectors – which determined the types of finance each could access and the type of projects each would develop.

### Alignment of South Africa with the GCF

Accreditation rendered the DAEs into legitimate partners for the GCF, forging connections through which resources could ultimately flow. However, for these relational ties to become productive, the DAEs needed to propose and legitimate investment opportunities. Each took a different approach. The DBSA quickly defined a narrow, clear scope that would only enrol components into the assemblage necessary to get bankable projects off the ground. Conversely, SANBI opened a call for proposals (CFP) to crowd-in partners and ideas, potentially creating more complex entanglements with a multitude of elements within the South African strata.

The DBSA expediently advanced several projects, including the CFF (GCF, n.d.-a). The CFF had the ambition of enacting a ‘paradigm shift’ by crowding-in private investment for projects with a climate rationale – an approach consistent with the GCF's own conception of transformative (see GCF, n.d.-a). The project blends GCF finance with DBSA resources and those of commercial banks to enhance credit terms for off-takers. An underpinning logic, framed around risk-weighted returns, is that lower interest rates and longer tenor on loans will encourage green investment. The development of the CFF hinged on the incorporeal transformation of economic sectors into tangible private sector investment possibilities. This was accomplished via a market study that quantified need and opportunity in terms of climate rationale and financial viability, fixing the project content. Commenting on the process, an author of the market study remarked:

*I was responsible for what a 10 billion Rand intervention would mean in the form of technology, carbon emissions reduction and employment. We have a water team, a waste team, an agricultural team, an energy team. We’ve got market intelligence. All our information comes from to-date information, from over 1760 members.*

Sector-specific information informed the study, conveying techno-economic credibility and authority (Sareen, 2022). This substantiated the rendering of the private sector as calculable to the GCF, creating a coherent, investible subject. It sought to make the market known and safe for investment, assuaging concerns about co-financing or sustained returns. It clarified the type of activity that would help realise the desires of the assemblage. This legitimated the project – in a promissory sense of future returns – where credit enhancement became the rubric of connection and the basis for productive relationships and future results with specified potential private sector partners.

The CFF is based on green bank principles (Orozco, 2019), targeting market gaps, and driving investment in support of South Africa's Nationally Determined Contribution (NDC). It uses public funds (the GCF's and its own) to leverage private investment at ratios ranging from 2:1 to 10:1, depending on sector and project specifics.^7^ The ratios derive from working knowledge of the economy and are vital to the GCF, which “wants to take one dollar and turn it into ten” (Commercial banker). The GCF must scale up its resources: marking the CFF out as a viable project.

The market study provides concrete details of the scope for investment in six sectors. For example, it is stated that current ZAR750 million market in commercial and industrial energy efficiency could potentially grow to ZAR480 billion by 2030.^8^ Sector breakdowns, combined with leverage ratios, constitute linguistic transformations of the economy – which remains unchanged – into economic opportunities. This can be understood as an exercise in what Nel (2017) describes as ‘speculative virtuality’, wherein conjectures of future benefits are leveraged to cohere the assemblage. The GCF is under pressure to disburse resources and generate results. The CFF offered a route to safely mobilise a large tranche of finance. The market study forged a connection between the GCF and the assemblage's core objectives: the circulation of resources, legitimating off-takers for concessional loans. It unleashed flows of resources and was a principal factor in the territorialisation (i.e., the ‘coming together’) of the project. The legitimacy machine works to incorporeally transform – that is, expressively change the status of – the economy from a set of challenges into a series of high-value opportunities, legitimating it as an investment option for the GCF. In turn, this empowers productive connections, enacting relational power.

The legitimacy machine likewise sought to incorporeally transform adaptation needs into grant finance options via SANBI. SANBI organised its project development around an open CFP to crowd-in ideas and concepts to produce legitimate options. This lengthy process included central government ministries and the national designated authority (NDA). SANBI held awareness-raising events to widen the scope for potential partners. The complexities of the South African response are compounded by a commitment to inclusive and participatory development. As a SANBI staff member remarked:

*We defined our niche and built the capacity of institutions to apply. Now this is tricky because who can apply for a GCF project? Is it only someone who is from an institution with a track record to manage a USD10m project? How is that transformative, especially in a country like South Africa where if you only work with privileged or the only ones who will benefit are privileged?*

Legacies of exclusion and inequality shaped this approach which sought to address uneven privilege (Baker and Phillips, 2019). Pursuing direct access saw SANBI sacrifice quick results in a bid to empower local participation and build capacity to govern climate change. SANBI's CFP was underpinned by an ambition to build a legitimate process with domestic stakeholders, who constituted its key legitimacy-granting audience. The CFP was effectively more legitimate than strictly necessary. SANBI could have established legitimate *enough* connections earlier with would-be beneficiaries to advance project development. According to a GCF Board Member in February 2020, that “money is there, we are waiting for them”. SANBI's drive to be participatory, just and legitimate saw a deep engagement with the people, ideas and things that would potentially be incorporated into the assemblage as adaptation investments. Its inclusive approach yielded multiple opportunities, enrolled potential collaborators, and built broad ownership, but resulted in large volumes of submissions of varying quality.

### Managing challenges and extending opportunities

Proposals were shortlisted and reviewed by a committee of sector experts, drawn from government ministries who struggled to prioritise projects. Asked how it achieved consensus, a Steering Committee member recalled how:

*(Laughs) We put the projects on a board, and we had round little stickers. Instead of making an open discussion, you just took a sticker and stuck it there, so the decision was made by counting the number of stickers because we were talking non-stop, and we were not progressing.*

The pragmatic use of stickers removed deliberation, broke deadlocks and maintained the flow of the assemblage, producing sufficiently legitimate outcomes. It established connections whilst pre-empting potential destabilising criticism. However, for many years this approach only yielded project concept notes, rather than full proposals. It narrowed options but the content remained in flux, restricting productivity in the adaptation space.

A conventional reading of the situation, emphasising legitimacy as a forward-facing qualifying attribute, might suggest that SANBI did not need to keep building legitimacy. It already had enough. Attention to the South African strata – the layered, stabilised arrangements that the assemblage draws on – offers an explanation. It has proven challenging for SANBI to stabilise and fix projects in South Africa. Staff would joke about how straightforward the process is elsewhere. Whilst discussing their progress in the margins of a consultation, one of the climate finance team shared their exasperation about how “they just sign off on any project” in neighbouring countries. SANBI's approach was no less urgent than DBSA's. The difference is that SANBI sought to establish and enhance lasting, productive connections without risking its social licence to operate. As an official from SANBI noted:

*…you know, I can’t develop a project and then go work next door while all hell breaks out around why was that project selected and not others. We’re in-country and we’re here to stay. The relationships we have with the sector departments go way beyond this GCF work. They are our brothers and sisters in the execution of the National Development Plan.*

The legitimacy machine makes visible the extra work in accessing grants for adaptation. For SANBI, it was more important to get things right than to move fast. SANBI needed to enrol a wide range of its partners and maintain legitimate connections as any challenge could delegitimate the assemblage and curtail its productivity. SANBI holds the keys to multiple flows of adaptation finance. Triggering short-term flows of resources by unlocking GCF finance might look good but could have compromised SANBI's ability to drive future change. SANBI was therefore cautious, reflecting the challenge in connecting its constituency with the GCF.

The CFF established a long list of options and was productive precisely because it crowded in private finance around vast potential interest in the money. By contrast, SANBI needed to work hard to equitably deny access to most interested parties. Grant finance for adaptation is acutely scarce, whilst private finance is vast and can be opened by initiatives like the CFF without excluding anybody. SANBI is accredited for projects worth up to US$50 million which is nowhere near the need in South Africa. Yet for SANBI's project development to be legitimate with its South African constituency it needed to extend this opportunity to projects it had no real prospect of funding. As such, it is straightforward to legitimate the economy as a set of opportunities, but difficult to narrow a limited opportunity for only a ‘deserving’ few. The legitimacy machine conceptualisation helps clarify the varied productivity of the DAEs in this way. SANBI was slow, resisting pressures to fund ‘any old project’ on account of it being harder to effect an incorporeal transformation of projects as legitimate without perpetuating legacies of exclusion (Essex et al., 2024).

Stabilising adaptation solutions was further complicated by discerning and engaged would-be beneficiaries, reflecting decades of experience in development cooperation. For example, an NDA staff member recounted that:

*We went to a community that wanted a different project to what the Fund could offer because it was trying to fuse in rainwater harvesting, climate smart agriculture and this community did not want anything to do with rainwater harvesting because in their mind this is just to bring in the JoJo tanks.*

JoJo tanks are water storage tanks, common in South Africa. Here, they symbolise one-way, passive development that ‘gives things to’, rather than partners with, communities. The quote emphasises SANBI's task to involve all its partners from the government through to communities. This is compounded by suspicion and fatigue amongst would-be beneficiaries:

*…outsiders must stop this Father Christmas syndrome – Father Christmas comes with this sledge, loaded with presents.*

(Community member and activist)

Many of the CFP concepts could have been adequate GCF projects. Fund accreditation went a long way towards empowering SANBI to do this. Whilst SANBI could have proposed a project sufficiently legitimate in the eyes of the GCF, doing so could have risked its privileged relationship with stakeholders should the project have disappointed. This is an example of SANBI's deep engagement where the desire to remain a trusted partner trumped that for GCF project approval.

The legitimacy machine established the necessary legitimacy to forge powerful connections through both DAEs. The DBSA needed only to transform the economy into private sector opportunities to animate these connections and trigger flows. It did what it needed to build *sufficient* legitimacy to mobilise finance. SANBI similarly fixed opportunities but faced a harder job in deciding which connections to animate. As such, it spent longer deciding which options to take forward as projects, stalling the assemblage's productivity. SANBI pre-empted a future legitimacy crisis by forging more legitimate connections than it could then use. It needed to build this broad legitimacy for its subsequent prioritisation to be legitimate. Through its strategically important relations with South African civil society, SANBI was somewhat hamstrung by the need to maintain legitimacy with deserving publics – communities it would ultimately have to disappoint due to the shortage of adaptation finance. Table 2 provides a comparative summary of the work of DBSA and SANBI.

**Table 2.** Comparing the work of the two DAEs.

Phase	DBSA	SANBI
GCF accreditation	DBSA transformed into a legitimate partner.	SANBI transformed into a legitimate partner.
Alignment of South Africa with the GCF	The South African economy is rendered an investment opportunity and legitimated as such to the GCF. Process was built on existing work in renewable energy procurement, experience with the South African Green Fund and private sector demand, private sector market study and experience with the REIPPPP. Enrolled the private sector, establishing the conditions for relational power.	Multiple South African adaptation options were advanced but not stabilised as investments. Process built on experience as accredited entity for the Adaptation Fund and ecological management function in South Africa. Call for proposals enrolled National Line Ministries and civil society leading to a wide range of potential partnerships and establishing the conditions for relational power.
Managing challenges, extending opportunities	The DBSA worked closely and expediently. The rubric of credit enhancement maintained legitimacy with a narrow set of actors (commercial banks, etc.) whilst maintaining distance from CSOs’ critique.	SANBI operated an inclusive project development process, maintaining a broad legitimacy. It did not foreclose connections and struggling to fix specific legitimate solutions to develop concept notes into full bankable GCF projects.
Exceeding the limits of the assemblage	DBSA pursued a wider objective of green bank expansion, where GCF projects could legitimate the DBSA to new audiences and access new sources of finance.	SANBI's approach to the GCF continued to prioritise its wider role and social licence to operate, slowing project approval.

Source: Authors.

The DBSA's management of potential challenges from domestic stakeholders and the GCF helps explain its productivity. The CFF's unifying rationale of credit enhancement meant that contracting parties (i.e., the commercial banks and off-takers of loans) need only buy-in to this rather than to project specifics. Relatedly, the DBSA maintained that they did not need to consult on the CFF because it is a facility, and the eventual sub-projects will be subject to consultations. Moreover, the off-takers of loans would not be the DBSA's clients, but those of the commercial banks. As such, responsibility could be passed on and furthermore deferred. The DBSA was thus expedient and fast, extending and maintaining productive connections, whilst excluding some actors who might otherwise slow processes of assembly. Through its strategic production, control and deployment of legitimacy, it was able to mobilise relational power to achieve its goals.

Indeed, DBSA's approach insulated its projects from critique. For example, in February 2019, the DBSA submitted another project to the GCF. This was an embedded generation project based on the Renewable Energy Independent Power Producer Procurement Programme (REIPPPP). Civil society organisations (CSOs) submitted written concerns to the Global CSO Representative, who presented these to the Board. These related as much to process and consultation as to project characteristics. The Board noted these concerns before promptly approving the project. Domestic CSOs were not considered direct stakeholders (either by the GCF or DBSA), and their critique did not impact what the project could deliver. CSOs directly challenged the legitimacy of the DBSA and its project development to the GCF, which had no bearing on the production of legitimacy.^9^ The episode contrasts with SANBI's efforts to maintain legitimacy through stakeholder engagement.

Another distinctive feature of the DBSA's expedient approach has been the separation of off-takers of the CFF loans from the politics of the assemblage via commercial bank intermediaries. The DBSA directly partners with commercial banks which serve as conduits for sub-projects, carrying out screening and client management:

*…deals come through the banks, through a screening process, the bank is not going to put forward credit or an opportunity unless they’ve vetted it. They’re doing a lot of the due diligence, so it is unlike an investor coming to DBSA to use the CFF, it is the banks coming.*

(CFF origination lead, DBSA)

The DBSA published a request for information and proposal templates that distil the precise needs of the GCF into a format that commercial banks populate. These documents strategically distributed agency by refining and aligning off-takers around credit enhancement to realise the potential in the market study. The future results promised by the CFF enact a speculative virtuality by promising credit terms, leverage ratios and climate impacts. This is fixed, and the deal sourcing with the commercial banks is an effort to fulfil those promises and realise the potential communicated in the market study. This is an example of the flows triggered by relational power, once the connections are legitimate.

### Exceeding the limits of the assemblage

The legitimacy machine changed the status of component parts and relations, making them sufficiently legitimate to forge productive connections. This in turn advanced flows of resources and, eventually, results. The assemblage is one of many and its intersection with others further clarifies each DAE's strategy.

Despite the DBSA's success in securing projects, by late 2019 their progress stalled.^10^ The DBSA is on a journey towards becoming a ‘green bank’, a desire which exceeds its GCF ambitions. The GCF programme was partly intended to demonstrate the DBSA's credentials, where the impact of the projects would legitimate the Bank for other opportunities. Impact evaluation provides an insight into the DBSA's journey. The DBSA included the CFF in the GCF's Learning-Oriented Real-Time Impact Assessment (LORTA). The assessment programme deploys mixed-methods evaluation techniques to assess outcomes and impacts of GCF-funded projects. Consultants working for the GCF visited the DBSA in November 2019 to plan the evaluation and visit potential off-takers of the CFF. LORTA requires a clear counterfactual (i.e., what financing would have been mobilised in the absence of the project) which proved elusive. A compromise was reached to assess impact in terms of the CFF's contribution to climate mitigation and adaptation goals. This so-called ‘climate rationale’ is a core outcome for GCF-supported activities. However, because LORTA could not measure the impact of the CFF in catalysing private investment, the findings could not be used to build a promissory legitimacy for the DBSA as a green bank in other contexts. The assemblage succeeded in producing legitimate subjects, processes, and projects before the throughput and coherence ebbed. This was because the assemblage struggled to deliver the larger goal: demonstrating impact. This higher-order purpose – beyond securing project funds – may be met with other partners, which means the DBSA has increasingly looked to other climate finance sources.

The establishment of GCF projects served the DBSA's strategic goal of green bank expansion. Legitimacy produced through this process was intended to trigger future opportunities. This is a requirement of the GCF, which is not intended to provide concessional finance and credit enhancement in perpetuity. The DBSA strategically manipulated legitimacy such that projects could be expediently approved, initiating access to new sources of concessional finance. SANBI has been more inward-looking, more concerned with its status as a trusted partner in South Africa. We summarise these insights in Table 3.

**Table 3.** Summary of results.

Stage	What does the legitimacy machine do?	What happened in practice?
Accreditation	The assemblage enrols multiple components to connect South Africa and the GCF. Accreditation incorporeally transforms each DAE into a legitimate partner that can access the GCF.	Each DAE accredited based on past experience, capacities and commitments to certain improvements. Accreditation ‘locked in’ each to certain pathways in terms of the type of finance they can access and what they can do with it. Each was subsequently able to submit projects to the GCF for approval.
Alignment of South Africa with the GCF	Each DAE seeks to incorporeally transform (i.e., change the status of) aspects of South Africa from problems into investment opportunities. This leads to legitimate components and relations that can form the basis of GCF projects.	The DBSA transformed the economy into a series of opportunities via the CFF market study, where high demand marked it as a good project for the GCF. SANBI solicited project ideas from would-be beneficiaries. High demand created a challenge in fairly allocating scarce adaptation funds.
Managing challenges, extending opportunities	Once potentially productive relations have been established, there is a strategic decision as to whether to enact this potential. DAEs can animate connections between legitimate component parts and relations, enacting flows of resources and, ultimately, results.	DBSA forged opportunities with commercial banks and the private sector. The rubric of credit enhancement connected stakeholders and the DBSA outsourced some decision-making and client management. Concerned with inclusion and fairness, SANBI opted to pursue a slower process with broad participation and buy-in, in order for difficult decisions about allocation to be accepted in South Africa.
Exceeding the limits of the assemblage	The assemblage is an ordering of human desire to access GCF finance. This is constantly in flux, and the assemblage may reorient around different desires. When this happens, the legitimacy machine orients around new problems and desires, enrols new components and seeks to transform these into legitimate ones.	The DBSA reorients around different sources of concessional finance, having in part been motivated to access the GCF to demonstrate its capacity to do this. SANBI remains committed to accessing the GCF, but this intersects with competing desires about its social licence to operate in South Africa and legitimacy with civil society.

Source: Authors.

## Discussion

A key theoretical innovation in this paper is to introduce the legitimacy machine and conceptualise legitimation as a strategic form of incorporeal transformation (Kumar, 2021; Suchman, 1995). Our conceptualisation takes us beyond thinking of legitimacy as a simple, static property possessed by actors (Bennett and Satterfield, 2018). Specifically, it emphasises the desires, processes and agency involved in the purposeful making and re-making of legitimacy. Moreover, it foregrounds the productive potential of legitimacy and its role in territorialising policy assemblages – in the sense of enrolling, ordering, and cohering different component relations to generate policy outputs and effects. Our legitimacy machine conceptualisation reveals how legitimacy can be actively enrolled and manipulated as a relational asset to create new possibilities (Stanley and Cusworth, 2025).

We show how legitimacy helps build, authorise and animate the connections that potentially enact relational power. Legitimacy unblocks and triggers flows of ideas and resources. It sustains the momentum and throughput of the assemblage in service of the core desire for project approvals. In making this claim about what legitimacy ‘does’, we are not seeking to essentialise legitimacy, positioning it as a necessary condition for the enactment of relational power. Indeed, in other assemblages, relational power may be produced and reproduced in other ways (Lawhon, 2012; Natarajan et al., 2019). However, to the extent that policy assemblages are often sites where authorisation, consent and credibility are necessary to ‘bring things together’, it is plausible that legitimacy may be an enabling resource for unlocking relational power in other settings (e.g., see Andres et al., 2023; Perkins, 2021; Vasstrøm and Lysgård, 2021). Our reading of legitimacy challenges Lau et al. (2020: 3) who theorise legitimation as a form of relational power that influences “access and exclusion to resources”. The assemblage-based analysis presented here suggests that legitimacy does not constitute power as such. Instead, legitimacy helps explain why certain relationships and associations *enable* power to function, flow and have material effects.

Our conceptualisation of the legitimacy machine has affinities with Ferguson's (1994) ‘anti-politics machine.’ Both can be understood as instrumental arrangements that serve human desires. Both machines have effects in the world. Like the anti-politics machine, the legitimacy machine may depoliticise development, using technocratic discourses to obscure thorny political issues and ‘get things done’. The legitimacy machine may similarly have effects in terms of reproducing institutional arrangements; in the present context, the growing dominance of profit-centred financial actors in climate finance. However, rooted in assemblage thinking, our rendering of the legitimacy machine offers a far more nuanced, contingent perspective than the (predominantly) Foucauldian-inspired anti-politics machine. Whereas Ferguson's account conveys an almost mechanical inevitability to the workings of a ‘development apparatus’ and its consequences, there is nothing automatic about the legitimacy machine. More specifically, underscoring the strategic nature of policy assemblages and the importance of heterogeneous desires, our analysis shows why actors might sometimes purposefully elect not to enact their productivity, and why. They may wish to protect or insulate themselves from legitimacy-diluting associations and outputs.

A focus on legitimacy also helps (re)centre politics into assemblage thinking (Kirkegaard et al., 2023; Richmond, 2019). It reminds us of the different, potentially contradictory, interests, goals, and logics that characterise assemblages (Barnes, 2022a; Köhne, 2014; Vasstrøm and Lysgård, 2021). It goes further by revealing legitimacy's dialectical role in (re-)politicising and depoliticising (Bond et al., 2019). On the one hand, the requirement for legitimacy can lead to new actors, interests, and rationalities being enrolled into the assemblage, opening-up governance processes to contestation, negotiation, and change. On the other, legitimacy can be a vehicle for depoliticisation, in that it can be acquired, configured, and strategically incorporated in ways that purposefully (or otherwise) narrow the scope for critique (Ernstson and Swyngedouw, 2024). Our observation that assemblages can be cohered and rendered productive by ‘just about enough’ legitimacy is especially useful in this regard.

The legitimacy machine clarifies and explains the varied productivity of two distinct DAEs. SANBI's desire for project approval and resources was secondary to its commitment to ‘slow and deep’ transformation. Our findings nuance research based on project proposals showing that many DAEs working on adaptation privilege speed over depth (Kuhl and Shinn, 2022). We moreover elaborate what this depth means in practice by demonstrating the connections that SANBI needed to manage to enact relational power. We show that SANBI remained committed to project development but stalled its productivity due to other desires. This demonstrates an important tension between speed and depth affecting organisations pursuing locally led adaptation (LLA) – where resources, ownership and decisions are devolved (Yee et al., 2025). Patience is at the core of LLA. SANBI's pace of project development cannot be explained away by capacity constraints or inefficiency. Inclusivity is shown to be slow and deep – a drawn-out process of building a common and shared legitimacy for the eventual use of finance. This is necessary given the normative importance and acute scarcity of climate justice finance (Bryant and Webber, 2024).

For the DBSA, credit enhancement, whereby every party gets some concession that they need or want, incorporeally transformed the economy into bankable opportunities for capital (Yunita et al., 2023). The body is unchanged: it is the same economy, energy infrastructure and financial sector. What has changed is the link forged around the rubric of credit enhancement that legitimates the CFF, making possible flows of resources and in turn, flow of results. The DBSA worked differently to SANBI and at times was criticised for its limited consultation. However, like SANBI, the DBSA did what it had to in order to sufficiently legitimate the component parts and relations of the assemblage. The Bank expediently forged powerful connections, legitimated by credit enhancement. This, in turn, facilitated the circulation of private capital necessary to fund the energy transition and other priorities. These activities are post-political, in the sense that there is a clear consensus in South Africa on the need for both external and domestic money to fund the energy transition (Barnes, 2025). We learn two things from this analysis. First, the DBSA was exemplary in terms of delivering a project that can catalyse private sector investment. The CFF addresses the urgent demand for finance as set out in South Africa's NDC. It is replicable and scalable, where new sources of finance can recapitalise the CFF, beyond GCF involvement. Second, our analysis cautions against simple comparisons between the two South African DAEs, helping us see beyond superficial assessments of effectiveness.

Our analysis also shows how each DAE reached a limit in the assemblage, where SANBI considered its wider licence to operate and the DBSA began to look beyond the GCF, to new lines of credit. The emergent nature of assemblages demonstrates how in each case, the desire to access the Fund is contingent and in a constant state of becoming. This finding can improve understanding of the less visible work of entities in the gap between accreditation and project approval. We have shown how these relationships remain in flux, with actors strategically looking to forge new connections. A benefit of an assemblage approach is evident when we think about these limits and the changing configuration of desire and strata involved in the DBSA's shift in strategy. We could equally imagine other limits, should climate change suddenly either stop being a concern or should sufficient climate-aligned finance begin flowing without the need for concessional support.

## Conclusion

This paper seeks to open the black box of GCF project development by offering an explanation for the uneven production of climate finance in South Africa from a multilateral climate fund. We foreground the importance of legitimacy in this endeavour, situating it as a strategic resource that actors mobilise and protect for different ends and for different audiences.

By focusing on the messy and non-linear process of project development, our account provides a sympathetic critique of the contrasting DAEs’ work. It would be easy to criticise the DBSA for a lack of engagement with national stakeholders and rushing through exclusive projects. Meanwhile, SANBI delayed access despite apparently being ready to submit a bankable project years before it eventually did, in 2025. An assemblage-based analysis – forged around our theorisation of the legitimacy machine – offers insights into how and why each entity worked the way it did. It shows how each responded to the different expectations and opportunities associated with the type and end use of the finance they sought. The DBSA worked expediently to crowd in private finance by legitimating the South African economy as an investible opportunity, and by insulating projects from critique by decentralising responsibility for consultation to commercial banks. It strategically mobilised sufficient legitimacy to progress project development through approval. SANBI worked slower, to include diverse views from civil society. Moreover, owing to the scarcity of adaptation finance, SANBI had to make difficult decisions about which projects to fund in order to secure its own legitimacy. It is understandable therefore that SANBI's inclusive approach to project development took considerable energy, political acumen, and ultimately, time.

Our findings speak to debates about international climate finance, nuancing critiques of the transformative impact of the GCF. Emphasising more critical conceptions of transformational, as opposed to the GCF's, we reveal how analysis of project documents and board deliberations may provide only a partial picture of the effects of GCF financing (Kuhl et al., 2024; Kuhl and Shinn, 2022; Puri et al., 2022). Specifically, by following project development processes, we show the potential for GCF projects to be transformational in a participatory sense and responsive to bottom-up desires. SANBI's slow, inclusive and deliberative approach contributed to better understanding of local adaptation needs – paving the way for projects that address underlying vulnerabilities. Crucially, it enrolled other executing agencies from the outset, increasing the likelihood that the project will deliver on its transformational potential through proper implementation. We suggest that SANBI is no outlier. Future research could usefully uncover more untold stories of country ownership (Barnes, 2022b) and DAEs’ efforts to forge legitimacy around contested opportunities. It could also go beyond the project development and approval process to explore the conditions under which the transformative promises of multilateral climate finance projects unfold in practice (Eriksen et al., 2021; Mills-Novoa, 2023).

We nuance critiques about financialization in international climate finance (Bracking, 2015; Bryant and Webber, 2024). The DBSA's approach exhibited many of the hallmarks of neoliberal financial logics, including an emphasis on mobilising private finance, safeguarding investor returns, and a preference for scalability (Baker, 2022; Bracking and Leffel, 2021; Grafe et al., 2025). Yet, foregrounding domestic agency (Hilbrandt, 2025), our analysis reveals that this was not imposed as ‘top-down financialization’ on the South African economy (Bertilsson and Thörn, 2021). Instead, the DBSA is shown to overcome barriers to accessing finance in a global system where African organisations remain marginalised. The DBSA responded to shortages of climate finance and the desire to crowd in private money for the energy transition, with the resulting debt almost exclusively taken on by private firms rather than socialised among public entities. These desires resonate with global agendas, to ‘turn billions into trillions’, but they are not forced on South Africa. We demonstrate how different actors are productive in different ways, reflecting the multiple pathways required to advance domestic climate agendas. The CFF fits the GCF's conception of ‘paradigm shift’ by legitimating and empowering the DBSA to access further sources of finance for climate projects in South Africa. Our work therefore serves as a corrective to accounts of international climate finance that conflate mobilising private capital flows with weak country ownership (Kalinowski, 2024). It also highlights the dangers of over-generalising narratives about the processes and effects of international climate finance.

Indeed, an important takeaway of this research is pragmatism and differentiation. The diverse needs for different types of finance require both rapid and patient approaches. This has policy implications for the GCF. The GCF and other actors in this policy space could further differentiate the policies, tools and demands for different forms of climate finance. Aligned with more pragmatic calls (Kreibiehl et al., 2022; OECD, 2025), they could increase efficiency through programmes that leverage public money to quickly mobilise private finance at scale. At the same time, there is an urgent need to provide additional support, flexibility and patience to national DAEs seeking grants for adaptation in ways that emphasise participation, deliberation and self-determination (Webber et al., 2022).^11^ The GCF has faced pressures from a range of actors – such as donors, potential recipients, and civil society organisations – to disburse funds quickly. Discourses of urgency have been mobilised to justify ‘fast and decisive’ action (Saddington, 2025: 448). However, our findings caution against these financial temporalities, whose effects in constraining more participatory forms of climate finance have been documented elsewhere (Diezmartínez and Short Gianotti, 2024). The GCF has committed to fund between 40–70 adaptation projects including LLA between 2024-27 (GCF, 2023: 6). As part of its ambitious agenda, the Fund should resist the impulse to exclusively move fast. To this end, it should acknowledge, promote and learn from the slow and deep transformative work exemplified by SANBI, embracing a more indeterminate temporality. The Fund can support similar DAEs and promote others to partner with them by better understanding the pressures and complexities of navigating and fixing legitimacy in the adaptation space. This might improve the experience of organisations like SANBI and encourage more such organisations to partner with the GCF. It might also help the GCF itself maintain its own legitimacy within a context in which the Fund faces questions about its ability to deliver on transformative socio-ecological change while efficiently scaling-up climate finance (Amighini et al., 2022; Omukuti et al., 2022).

## Highlights

1. This paper explains the varied and uneven Green Climate Fund access in South Africa.
2. We introduce the ‘legitimacy machine’ as a heuristic device to advance understandings of how and why assemblages are productive.
3. Assemblage thinking is used demonstrate how legitimacy is a strategic resource, shedding light onto how relational power is enacted.
4. Acute shortage of grant finance necessitates slow and deep engagement to legitimate difficult allocation decisions.
5. Greater differentiation in the Green Climate Fund's operations could support both private and public sector transformation.

## Data Availability

The data for this study is not publicly available.

## Notes

1. As of the 43^rd^ Board Meeting in October 2025, the DBSA had three active projects and one that has lapsed without implementation.

2. Concept notes summarise proposed projects and are submitted to the GCF for feedback to support full project development.

3. It is important not to confuse ‘incorporeal transformation’ with the idea of ‘transformative’ or ‘transformational’ change used in the climate finance literature. Our argument is that processes of incorporeal transformation make things legitimate so that climate finance projects can be assembled. This does not imply transformational change in terms of decision-making processes, developmental impacts or structural change.

4. Bodies here refer to components of the assemblage, both humans and other ‘things’ such as policies, organisations and ecosystems.

5. The Global Environment Facility (GEF), another multilateral climate fund.

6. A partnership between development banks and not formally associated with the GCF.

7. From the market study, not publicly available.

8. From the market study, not publicly available.

9. Underlying this critique is a wider source of discontent, stemming from CSOs’ desire for GCF funds to be used differently, e.g., by SANBI for adaptation.

10. The DBSA secured another project in 2023, a private sector water scheme (see www.greenclimate.fund/project/fp209)

11. We thank an anonymous reviewer for this suggestion.
